# A paradox of local abundance amidst regional rarity: the value of montane refugia for Persian leopard conservation

**DOI:** 10.1038/s41598-019-50605-2

**Published:** 2019-10-11

**Authors:** Mohammad S. Farhadinia, Brett T. McClintock, Paul J. Johnson, Pouyan Behnoud, Kaveh Hobeali, Peyman Moghadas, Luke T. B. Hunter, David W. Macdonald

**Affiliations:** 1 Wildlife Conservation Research Unit, University of Oxford, Tubney House, Oxfordshire, OX13 5QL Oxford, UK; 2 Future4Leopards Foundation, Tehran, Iran; 3 Marine Mammal Laboratory, Alaska Fisheries Science Center, NOAA‐NMFS, 7600 Sand Point Way NE, Seattle, Washington, 98115 USA; 4 Wildlife Conservation Society, 2300 Southern Blvd, Bronx, 10460 USA

**Keywords:** Conservation biology, Population dynamics

## Abstract

The population densities of leopards vary widely across their global range, influenced by prey availability, intraguild competition and human persecution. In Asia, particularly the Middle East and the Caucasus, they generally occur at the lower extreme of densities recorded for the species. Reliable estimates of population density are important for understanding their ecology and planning their conservation. We used a photographic spatial capture-recapture (SCR) methodology incorporating animal movement to estimate density for the endangered Persian leopard *Panthera pardus saxicolor* in three montane national parks, northeastern Iran. We combined encounter history data arising from images of bilaterally asymmetrical left- and right-sided pelage patterns using a Bayesian spatial partial identity model accommodating multiple “non-invasive” marks. We also investigated the effect of camera trap placement on detection probability. Surprisingly, considering the subspecies’ reported low abundance and density based on previous studies, we found relatively high population densities in the three national parks, varying between 3.10 ± SD 1.84 and 8.86 ± SD 3.60 individuals/100 km^2^. The number of leopards detected in Tandoureh National Park (30 individuals) was larger than estimated during comparable surveys at any other site in Iran, or indeed globally. Capture and recapture probabilities were higher for camera traps placed near water resources compared with those placed on trails. Our results show the benefits of protecting even relatively small mountainous areas, which accommodated a high density of leopards and provided refugia in a landscape with substantial human activity.

## Introduction

Resource availability, notably prey density, is the main ‘bottom-up’ process affecting predator density^1,2^. Conversely, ‘top-down’ processes, such as disease^3,4^, human persecution^5,6^ and competition^7,8^ can also operate to shape predator populations. Many apex predators may impose top-down regulation on the density of their prey and smaller meso-predators^9,10^. Where they compete with larger predators, they are themselves subject to varying degrees of top-down regulation, manifested either in their behaviour^11,12^ or population density^8,13^ (but see^14^). The world’s iconic carnivores can be either apex or subordinate predators in different parts of their range. The common leopard *Panthera pardus*, one of the most wide-ranging top predators, frequently illustrates these dual circumstances.

These opposing regulatory processes, particularly environmental productivity have contributed to great variation in density estimates of leopards across their global range. Estimates of leopard population densities vary 150-fold (see Supplementary Table S1) from 0~0.1 individuals per 100 km^2^ in northeastern China^15^ to 14.9 individuals/100 km^2^ in north central India^16^. Leopards may reach particularly high densities in the absence of larger competitors^8,17^. However, while there are numerous studies reporting leopard density, studies from the Middle East and the Caucasus, where competition with other large felids is absent, are few^18,19^. Surveys from the protected areas of this region have reported densities at the lowest known extreme for the species, fewer than 0.5 individuals/100 km^2^ ^20^. Importantly, the few individuals observed in protected areas frequently show no evidence of breeding^20–23^. Low density and apparent lack of breeding are clearly grounds for conservation concern.

Precise population estimates are important for conservation actions and for monitoring their outcomes^24^. Camera-trap data and capture-recapture analyses is the method of choice for estimating the density of large and small cats with individually distinct coat patterns^25^. We employed spatial capture-recapture (SCR) methodology^26,27^ by means of motion-detector camera traps in three national parks in northeastern Iran to estimate population parameters of the endangered Persian leopard *P*. *p*. *saxicolor*. We adopted a recently developed statistical method for integrated mark–recapture analyses using bilaterally asymmetric photo-identification records which accounts for uncertainty about the true number of distinct animals observed in the study^28–30^.

Maximising the number of captures and recaptures is known to enhance the precision of estimates derived from this methodology. Attractants, such as bait^31^ and scent lures can increase capture rates^32^. However, they may also modify the ranging behaviour of animals and may cause the animals to move beyond their usual home range. Individuals can also change their behaviour in response to the first encounter event, which induces non-independence of encounter probability in the encounter history of that individual^33^. Attractants can also amplify individual heterogeneity in encounter probability as their effects may vary with age, sex or resident status (see^34^ for review). Therefore, our second objective here was to explore the use of a natural limiting factor which is not associated with these various disadvantages of attractants, i.e. water resources during summer on estimates of detection parameters and observed age/sex class composition in leopards. Spatial variation in density in areas where populations are likely to be low has implications for leopard conservation in this poorly studied area.

## Results

A total of 5410 trap nights (Table 1), provided a total of 1335 pictures containing 477 leopard detections, resulting in 44 independent individuals based on left flank markings (16 males, 14 females and 14 with unknown sex). Based on right flanks markings (see Methods for details) we identified 52 independent individuals (23 males, 20 females and 9 with unknown sex; Table 2).

**Table 1:** Details of sampling design for spatially-explicit capture-recapture framework across three study areas in northeastern Iran (2015–2016).

Study area	Area (km^2^)	# stations (# leopard positive stations)	Sampling period (days)	Season	Effort (trap nights)	# available grids (# sampled grids)	MCP CT stations (km^2^)	# stations/grid
Tandoureh NP	355.4	80 (50)	31.5 to 25.7.2016 (55)	Spring-Summer	3597	47 (39)	277.5	2.1 (SE 0.1)
Salouk NP & PA	199.1	22 (15)	20.10 to 19.12.2015 (60)	Autumn	1040	17 (11)	50.7	1.8 (SE 0.3)
Sarigol NP	70.4	19 (17)	22.10 to 16.12.2015 (55)	Autumn	852	10 (9)	38.4	2.1 (SE 0.3)
Total		121 (82)			5410	74 (59)		

NP = National Park and PA = Protected Area.

**Table 2:** Details of baseline information on leopards based on systematic camera trapping across three study areas in northeastern Iran.

Study area	# leopard pictures	# independent leopard detections (# non-identifiable)	# detected independent individuals			Sex composition	# families	# dependent cubs
			Right flank	Left flank	Both flanks			
Tandoureh NP	1097	354 (67)	30	26	21	15 M, 14 F, 1 U	5	7
Salouk NP & PA	99	56 (9)	12	10	7	4 M, 4 F, 4 U	1	2
Sarigol NP	139	67 (18)	10	8	4	4 M, 2 F, 4 U	1	1
Total	1335	477	52	44	32	23 M, 20 F, 9 U	7	10

To calculate the number of independent leopard detections, we discarded all but one capture of the same individual taken at the same camera station no more than 0.5 hours apart. Sex compositions, number of families and dependent cubs are based on right flank detections. NP = National Park, PA = Protected Area, M = Male, F = Female and U = Unknown sex.

The median number and range of captures per individual was 1.5 (1–18), 2.0 (1–15) and 3.0 (33) in Sarigol, Salouk and Tandoureh, respectively. Leopards in Tandoureh had a higher detection frequency compared with the other two sites (Z = 2.33, *P* = 0.02, Negative Binomial regression) with goodness of fit test (residual deviance = 53.67, df = 49, *P* = 0.29), indicating adequate fit. In Sarigol, Salouk and Tandoureh respectively we observed five, three and 12 individuals that were captured only once – these comprised between 25% (Salouk) and 50% (Sarigol) of the total independent leopard individuals.

We also detected seven leopard families with a total of 10 cubs (mean 1.4 ± SE 0.2, ranging 1–2; Table 2), based only on right flank detections. The cubs comprised between 9.1% and 19.4% of the total number of detected individuals in each area. In Tandoureh where we had multiple detections of some members of each leopard family (n = 35 for five families), cubs were photographed with the female only in 19 cases (54.3%) whereas the adult female was the only representative of each family in the rest of the detections (n = 16, 45.7%).

### Abundance and density estimation

For Sarigol, models including a constant detection probability accounted for 0.53 of the posterior model weight, while models including temporal trends (*˜Time*) and behavioural effects (*˜c*) represented 0.23 and 0.21 of posterior model weight, respectively (Table 3). In contrast, there was strong evidence of a decreasing time trend in detection probability for Salouk as the temporal trend model (*˜Time*) accounted for the majority of posterior weight (0.94) while the constant detection probability accounted for 0.04 of posterior model weight. In Tandoureh, there was strong evidence of additive behavior and trap placement effects on trap-specific detection probability (0.82 of posterior model weight for *˜c* + *Placement*) while the interactive model of camera trap placement and behavioral response to first capture (˜c*Placement) accounted for 0.18 of posterior model weight.

**Table 3:** Posterior model probabilities (PMM) for Persian leopards in northeastern Iran.

Model	PMM
***Sarigol***	
p(˜1) delta(~1)	0.53
p(˜Time) delta(~1)	0.23
p(~c) delta(~1)	0.21
p(~time) delta(~1)	0.03
***Salouk***	
p(˜Time) delta(~1)	0.94
p(˜1) delta(~1)	0.04
p(~c) delta(~1)	0.02
p(~time) delta(~1)	0.00
***Tandoureh***	
p(~c + water)delta(~1)	0.82
p(~c * water)delta(~1)	0.18
p(~1)delta(~1)	0.00
p(~time)delta(~1)	0.00
p(~Time)delta(~1)	0.00
p(~c)delta(~1)	0.00
p(~water)delta(~1)	0.00

Models for detection probability (p) included no effects (˜1), behavioural effects (˜c), time variation (˜time) and temporal trends (˜Time). In Tandoureh, three additional models were fitted as effects of camera trap placement (˜Placement), additive of camera trap placement and behavioral response to first capture (˜c + Placement) and interactive effect of camera trap placement and behavioral response to first capture (˜c*Placement).

In Sarigol, the constant detection probability model was supported by a model-averaged *p* of 0.16 ± SD 0.06 at the first to 0.12 ± SD 0.05 at the last sampling occasion (Table 4). The model-averaged *p* in Salouk ranged from 0.14 ± SD 0.07 to 0.03 ± SD 0.02 between the first and the last sampling occasion, supporting a decreasing time trend in *p*. In Tandoureh, at water resources, the probability of capture was 0.04 ± SD 0.01 while the probability of recapture was 0.36 ± SD 0.04. In contrast, it was 0.02 ± SD 0.004 and 0.19 ± SD 0.03 for the probability of capture and recapture, respectively, at trails.

**Table 4:** Model-averaged posterior mean, standard deviations and 95% credible intervals (CI) for models including only those covariates which received >0% of the posterior model weight for each area in northeastern Iran.

Parameter	Posterior mean	SD	CI
***Sarigol NP***			
*D*	8.86	3.60	2.00–16.65
*σ*	720	1200	200–4900
*σ*_Male_	2300	2590	190–8980
*σ*_Female_	620	1390	113–6020
*α*	0.79	0.16	0.40–0.99
*δ*	0.45	0.02	0.40–0.49
*p*_first occasion_	0.16	0.06	0.07–0.31
*p*_last occasion_	0.12	0.05	0.04 0.23
*c*_second occasion_	0.16	0.05	0.08–0.28
*c*_last occasion_	0.13	0.05	0.04–0.23
*Ψ*	0.80	0.11	0.55–0.97
***Salouk NP & PA***			
*D*	3.10	1.84	1.08–7.40
*σ*	3900	2300	950–9200
*σ*_Male_	4350	2290	1150–9390
*σ*_Female_	4340	2660	745–9590
*α*	0.87	0.10	0.63–0.99
*δ*	0.38	0.03	0.31–0.43
*p*_first occasion_	0.14	0.07	0.05–0.32
*p*_last occasion_	0.03	0.02	0.01–0.08
*c*_second occasion_	0.12	0.06	0.05–0.28
*c*_last occasion_	0.03	0.03	0.01–0.09
*Ψ*	0.79	0.10	0.56–0.95
***Tandoureh NP***			
*D*	5.57	1.04	3.74–7.80
*σ*	2000	500	130–3200
*σ*_Male_	3290	1460	1770–6960
*σ*_Female_	920	210	615–1420
*α*	0.91	0.03	0.84–0.96
*δ*	0.31	0.02	0.28–0.34
*p*_Water_	0.05	0.01	0.04–0.06
*p*_Trail_	0.02	0.004	0.02–0.03
*c*_Water_	0.36	0.04	0.30–0.43
*c*_Trail_	0.19	0.03	0.14–0.24
*ψ*	0.88	0.06	0.75–0.96

*D* = population density in independent leopards per 100 km^2^, *σ* = distance term for the detection function (km), *α* = conditional probability of a simultaneous type 1 and type 2 encounter (given both mark types detected), *δ* = conditional probability of type 1 (left flank) or type 2 (right flank), *p* and *c* = probabilities of capture and recapture respectively, *ψ* = probability that a randomly selected individual from the *M* = observed individuals belongs to the *n* unique individuals encountered at least once. To investigate the effect of sex, the best performing model for each area as *mod*.*p* = *~c* + *Time* (Sarigol and Salouk) and *mod*.*p* = *˜c* + *Placement* (Tandoureh) were run for each sex separately.

The model-averaged posterior mean was *D* = 8.86 ± SD 3.60 (95% credible interval: 1.96–16.7) independent individuals/100 km^2^ for population density of leopards in Sarigol. In Salouk, the model-averaged posterior mean was *D* = 3.10 ± SD 1.84 (95% credible interval: 1.08–7.40) independent individuals/100 km^2^ whereas in Tandoureh it was estimated as *D* = 5.57 ± SD 1.04 (95% credible interval: 3.74–7.80; Table 4) independent individuals/100 km^2^. With constant *δ*_1_ = *δ*_2_, the model-averaged posterior means for *δ* were similarly high across the three study areas, showing that the conditional probabilities of both-flank encounters were relatively infrequent (Table 4).

There was no evidence of sex-based difference in *p* and *c* for all the study areas, based on the overlap between male and female 95% credible intervals (Supplementary Table S2). However, intersexual difference was seen in σ only for Tandoureh, as σ_Male_ (3290 ± SD 1460) was larger than σ_Female_ (920 ± SD 210; Table 4).

### Effects of sampling design in Tandoureh

**Figure 1:**
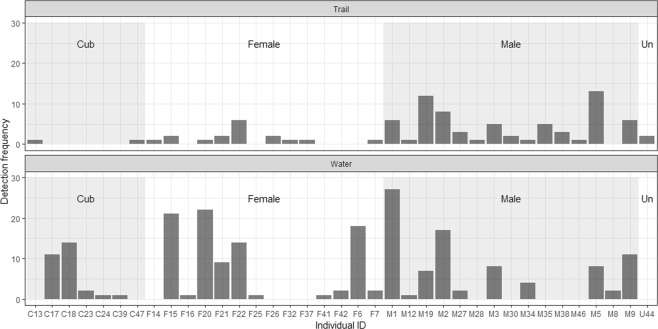
Comparison of detection frequency for all demographic classes between water and trail-based camera traps. Each code on the x-axis refers to a single individual leopard within the relevant demographic class, corresponding to images; M = male, F = female, U and Un = unidentifiable and C = cub.

Preferential water-based sampling increased detections per individual by 2.3-fold in Tandoureh (water-based = 7.9 ± SE 1.5 versus trail-based = 3.4 ± SE 0.7), but the number of independent individuals detected at water was slightly lower (20 on-water versus 24 on-trails, Fig. 1). In total, 9 independent leopards were detected only once on cameras placed at trails whereas it was only four individuals at water resources (Fig. 1).

There was no evidence that the total number of unique individuals detected from each age/sex group varied with sampling method (Fisher’s exact test, *P* = 0.40). The frequency of detection per individual, however, was higher for each age/sex group for water-based cameras (Fisher’s exact test, *P* < 0.001; Table 5). There was evidence that the effect of camera-trap placement on detection success varied among age/sex groups (interaction term X^2^ = 16.82, df = 3, *P* < 0.001). While all age/sex classes were more frequently detected at water-based cameras, this ranged from 1.7 times more in independent males (Z = −2.31, *P* = 0.29) to 4.7 times more in independent females (Z = −5.69, *P* < 0.001, Fig. 1 and Table 5). Cubs were detected mainly at water resources, both in terms of numbers and the frequency of detection per each individual cub (Fig. 1 and Table 5).

**Table 5:** Detection frequencies compared between trail and water-based sampling in Tandoureh NP.

Parameter	Camera trap placement		Water/trail ratio
	Water	Trail	
***Detection frequency*** (***SE***)/individual*			
Independent male	8.1 (2.4)	4.8 (1.1)	1.7
Independent female	8.9 (2.7)	1.9 (0.5)	4.7
Cub	5.8 (2.8)	1.0 (0.0)	5.8

^*^A single individual with unknown sex was excluded from sampling comparison.

## Discussion

We documented the highest densities of leopards in the Middle East and the Caucasus, as well as the largest number of leopards detected at any location surveyed to date. Our findings highlight the importance of northeastern Iran as a leopard hotspot, and thus a focus of conservation.

**Figure 2:**
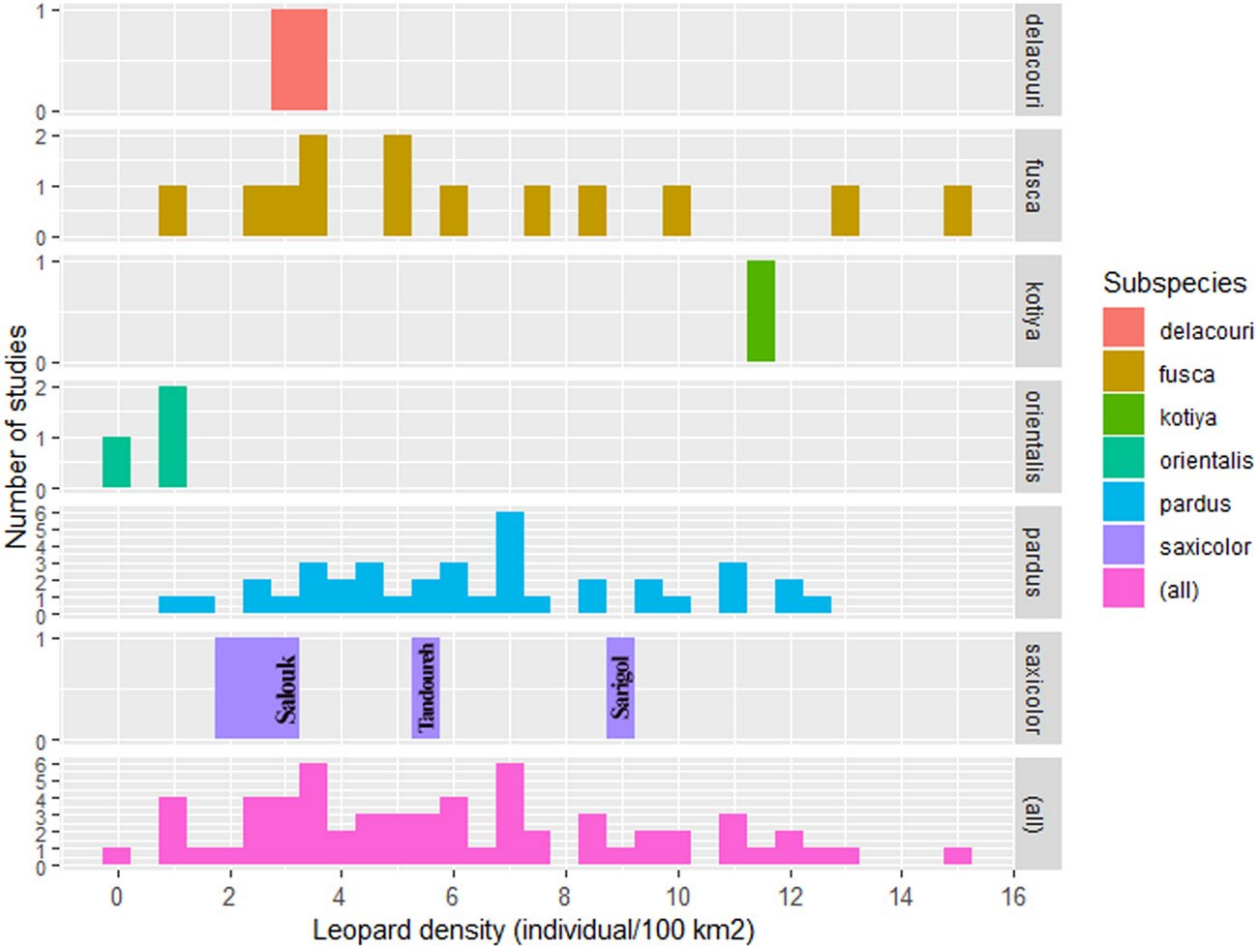
Distribution of density estimates for leopard subspecies across the species global range based on 72 published leopard estimates (see Supplementary Table S1).

Leopard density estimates in northeastern Iran far exceeded estimates made elsewhere in the Middle East and the Caucasus, ranging between 0.34 and 2.63 individuals/100 km^2^ ^19,20^. Our estimates were also higher than the majority of published leopard densities across the species’ continental Asian range (Fig. 2 and Supplementary Table S1) where higher estimates have been recorded only at a few protected sites in India^35,36^.

Dependent cubs have generally low capture probabilities in population studies of large felids^37,38^, and the same is true of leopards^31^. Attractant-based camera trapping, such as the use of baits, can substantially improve the detection of cubs^31^. We showed that using natural limiting factor, such as water resources, which do not have the disadvantages of other attractants (see Introduction for more details) increases the cub detectability. Although the proportion of cubs in the population in northeastern Iran was higher than almost all available estimates from different subspecies of leopards (Supplementary Table S1), mother leopards in almost half of detections were photographed without their cubs. Lower mobility of younger leopard cubs^39^, the female habit of hunting alone^40^ and the time lag between consecutive photo shoots needed to charge the Xenon flash^41^ may explain the lower detectability of cubs.

We found no evidence that detection probability (*p*) varied between sexes, unlike previous SCR estimates on leopards^32,36,42^ where wider ranging by males and potential avoidance of trails by females, which are frequently patrolled by adult males, resulted in higher male detection rates^24^. Sexual patterns of space-use among large cats are widely considered to differentiate SCR parameters and so influence density estimates and associated parameters^42–44^. Differences in habitat accessibility may explain the lack of a marked male-bias in our detection probabilities. Thus, it is less likely that leopards use steep cliffs for moving in the landscape in rugged terrain, using instead ridgelines and valley bottoms, resulting in similar detection probability for males and females. Similarly, the detection probability did not vary between the sexes in another montane big cat, the snow leopard *P*. *uncia*^45^. Forest or lowland landscapes, by contrast, may allow large cats to partition space use differently between the sexes, resulting in different detectabilities^32,42,46^.

Movement patterns have implications for density estimates, detection probabilities and the precision of density estimates^34,47^. Leopards show marked variation in their use of space, depending on age, sex^40^, reproduction status^48^ and season^49^. High detection probability and more precise density estimates can be achieved by conducting camera trapping surveys in summer (after a minimum of one month following the birth time when cubs have more mobility) and autumn (before dispersal), while reducing the likelihood of violating demographic closure.

The two sampling methods yielded slightly different numbers of leopard detected. There are two plausible reasons. First, the higher number of trail stations compared to water-based cameras (55 versus 25) and the spatial configuration of trail cameras which provided more coverage (72.3% versus 38.2% of sampling girds) resulted in more individuals detected along the trails than at water resources (24 versus 20). Second, cameras at water resources achieved both higher capture rate (0.05 ± SD 0.01 versus 0.02 ± SD 0.004 captures for individual) and recapture rate (0.36 ± SD 0.04 versus 0.19 ± SD 0.03 recaptures for individual; Table 4) as well as a lower number of individuals with single detections (4 versus 9 individuals).

The use of attractants such as scent lures or baits involves some extra cost. Arguably, this has a negligible effect on population density estimates^32^, but may slightly increase precision estimates for leopard populations^31^. Although almost similar numbers of individuals were detected at both trail-based and water-based samplings, the latter yielded higher frequency of detections with significantly lower operational costs. The water-based sampling can help to detect less detectable age/sex groups, particularly females and cubs, which widely remain undetected during camera trapping surveys in leopard areas (40.0% of published papers failed to detect any leopard cub; Supplementary Table S1). The concentration of cameras and leopards around water resources in hot months resulted in a higher recapture rate in Tandoureh, which is commonly seen in baited sampling surveys^31^. Future studies are encouraged to investigate the potential advantages and disadvantages of preferential water-based sampling on density estimation e.g.^50^, as well as potential inclusion of habitat covariates in the spatial point process model for the activity centres e.g.^51^. We also recommend deploying two camera traps per station to reduce uncertainty associated with individual identification, if funding and logistical constraints do not preclude this option given the geographic coverage required^34^.

We acknowledge two possible sources of positive bias in our density estimates. First, a trapping array smaller than the average home range can positively bias the density estimate^44,52^ due to temporary emigration^53^. However, SCR models can perform well for this scenario^54,55^, if the number of individuals detected is more than five^44^. As such, density estimates in Salouk and Sarigol, where at least 10 individuals detected in trap arrays smaller than the home range size, are unlikely to be affected to any important degree by this source of bias. Nonetheless, the high detection probability at home range centres and the small movement parameter in Sarigol suggest that the trap array has perhaps captured the edges of many home ranges^52^. Second, the effect of water resources as a limiting resource can vary seasonally. Therefore, leopards may shift their home ranges to areas close to water resources more intensively during hot months^49,56^, which can result in higher leopard densities, especially if individuals, which are normally not present in the study area, move into the area during hot months.

Eight Asian subspecies of leopards have experienced approximately 85% range loss^57^, and often now occur at perilously low densities (see Supplementary Table S1). Conservation interventions to halt, and reverse, these declines, and monitor recovery, require unbiased and precise estimates of population density. Our study provides a protocol for achieving this, while mindful of minimizing operational costs. This is particularly timely in the Middle East and the Caucasus, where several of the last remnant populations of leopards occur along international borders, such as Iraq, Turkey and Iran^23^, the Lesser Caucasus^22,58,59^, the Kopet Dag Mountains along the Iran-Turkmenistan borderlands^60^, and Yemen, Oman and Saudi Arabia^61^. Unfortunately, many of these areas are badly affected by military conflicts and security concerns, which are not currently attractive for conservation investment. Importantly, the Belt and Road Initiative BRI), linking China to Europe via land and maritime networks, traverse near key habitats for Persian leopards in northeastern Iran. As a potential threat to the region’s leopard populations, the BRI can create new supply sources for illegal wildlife trade to meet the demands of traditional Chinese medicine^62^.

The high population density of leopards observed in this study, contrary to expectation, illustrate that an area as small as the home range of a single individual^49^ can provide a refugium for a high density of leopards. Importantly, controlling two threats, prey depletion and leopard poaching are crucial to achieve this.

## Methods

### Study area

**Figure 3:**
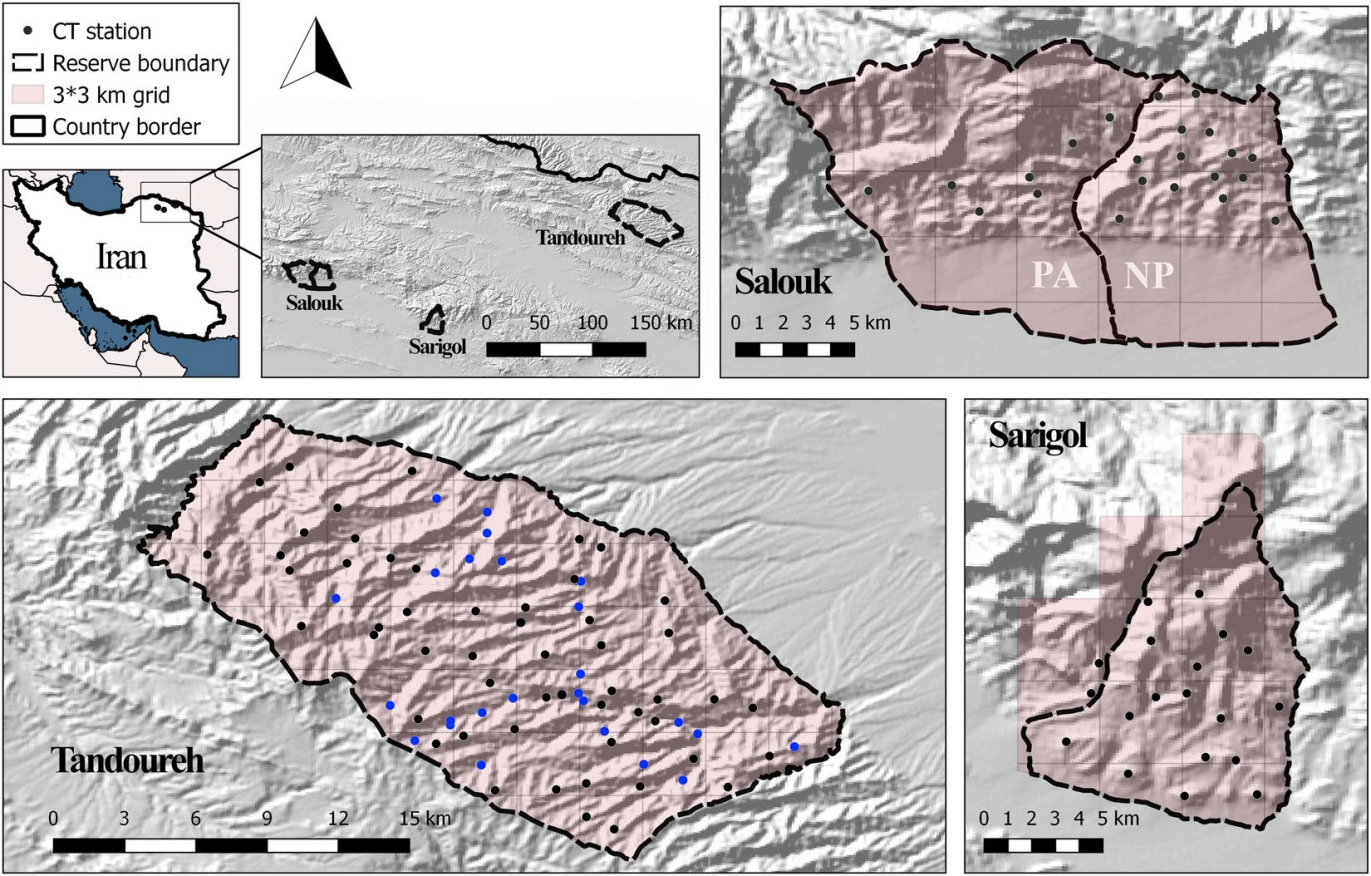
Spatial configuration of study areas and locations of camera trap stations across three reserves in northeastern Iran. The map inset shows locations of the study area in Iran. In all study areas, we conducted camera trapping surveys inside the national park, except at Salouk where we expanded our sampling to cover both National Park (NP) and Protected Area (PA). In Salouk, grids located in southern plains were not sampled and higher elevations were not accessible during the survey due to extreme weather conditions. Blue dots represent water-based camera trap stations. Maps were created using Quantum GIS software version 3.2.1 (QGIS Development Team, https://qgis.org/en/site/).

The Kopet Dag and Aladagh Mountains in northeastern Iran host a number of montane reserves, including Tandoureh National Park and Protected Area (hereafter NP and PA), Salouk NP & PA and Sarigol NP & PA, lying at the eastern extreme of the Irano-Anatolian Biodiversity Hotspot (E57°15′ to E59°15′, N36°20′ to N37°20′; Fig. 3 and Table 1). They total 930 km^2^ of very rugged mountainous landscapes with steep cliffs and deep valleys (Fig. 3 and Table 1) with the temperate semi-arid climate and mean annual precipitation of 200 to 300 mm^63^. Similar elevation range is seen across the three study areas, varying between 1000 to 3000 m.a.s.l.

The vegetation is generally dominated by scrub, particularly *Astragalus* spp. and *Artemisia sieberi*. Potential ungulate prey for leopards include urial *Ovis vignei*, bezoar goat *Capra aegagrus*, and Eurasian wild pig *Sus scrofa*^63^. NPs in Iran are subject to stringent law enforcement, and livestock grazing is completely banned. PAs, by contrast have lower levels of protection, and less intense anti-poaching efforts. Furthermore, nomadic pastoralists are permitted to graze their herds in non-NPs during summer (May-August). Wild ungulates such as bezoar goat and urial range within the boundaries of the reserves where anti-poaching measures are in place. In contrast, domestic ungulates graze outside the reserves where leopards occasionally attack stock^60,64^.

### Sampling design

The study was approved by the Iranian Department of Environment (research permit number 93/16270) and was performed in accordance with relevant guidelines and regulations. We deployed camera traps for 55 to 60 days within each area (Table 1), a survey effort which is adequate for obtaining reliable estimates^44^. Each survey period consisted of up to 12 sampling intervals, each lasting for five days.

Although SCR models relax the geographic closure assumption (the model allows for movements of individuals about their activity centres), SCR models do, however, assume demographic closure- no birth or mortality as well as no permanent emigration from, or immigration into, the state-space^47^. There are no specific population closure tests for SCR models, mostly due to the fact that behavioral variation in detection is indistinguishable from violation of demographic closure^33,47^. Therefore short time periods and incorporating the biology of species are necessary to avoid violating the demographic closure^34,47^. Hence, our survey period (≤2 months) was short enough to assume demographic closure, based on previous studies on leopards^24^. Equally important, we did not conduct camera trapping surveys during two seasons which can violate demographic closure^34^: (1) birth season peaked during mid-spring in northeastern Iran^65^ and (2) dispersal period of young leopards after independence which can happen at the age of around 19 months^39^ (i.e. late autumn onwards).

We deployed a mean of 2.0 (SE 0.2) camera trap stations on park-wide 3 × 3 km grids, all with a single camera (Fig. 3 and Table 1). The smallest home range size for a resident male from northeastern Iran is calculated as 63.3 km^2^ (minimum convex polygon)^49^. Based on a female/male home range size ratio of 0.4^31^, we assumed a minimum female home range size of approximately 25 km^2^, resulting in at least five camera traps within each leopard range. Camera stations were placed at a mean spacing of 1250 (SE 90), 1400 (SE 87) and 1220 (SE 63) m in Salouk, Sarigol and Tandoureh, respectively, in order to simultaneously achieve the twin objectives of maximizing the number of individuals caught and adequately recapturing individuals at different camera traps, as required in SCR designs.

**Figure 4:**
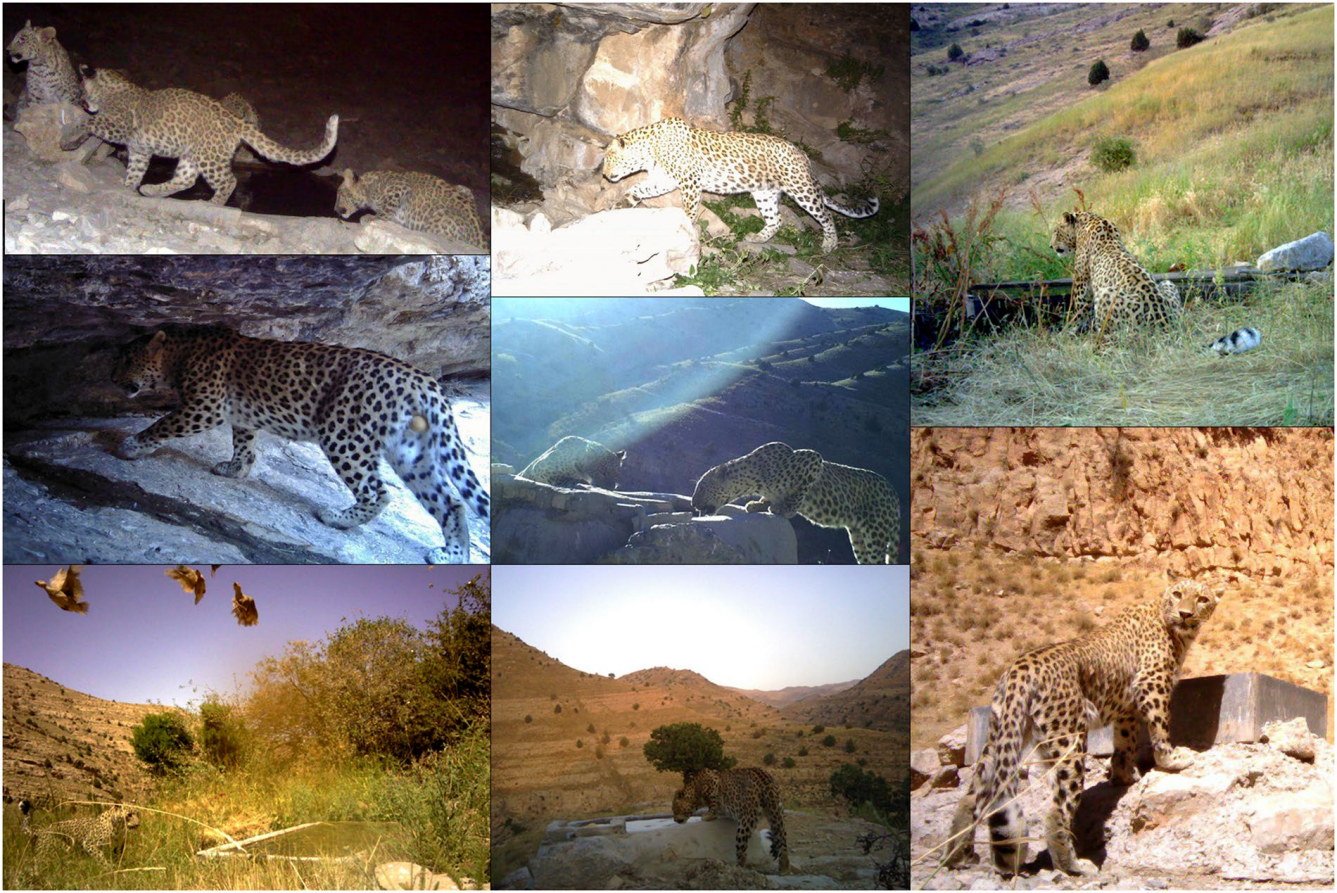
Some examples of Persian leopard photos at different types of water resources in Tandoureh National Park during summer 2016, northeastern Iran (© FLF/IranDoE).

In Sarigol and Salouk, camera traps were placed either along ridgelines (n = 34, 82.9%) or valley bottoms in autumn. In Tandoureh, where the survey was conducted during summer in the driest period of year (June to August), we established two sampling protocols to investigate the effect of camera trap placement on detection probability and observed age/sex composition. First, 25 water resources (springs or artificial waterholes, 31.3% of stations) were each equipped with a camera trap (Fig. 4). We did not place any camera traps on springs within marginal grids due to the risk of vandalism. Nonetheless, we are confident that approximately half of the national park’s known water resources were sampled using camera traps. Second, we also placed cameras along trails (n = 55, 68.7% of stations), predominantly along ridgelines in Tandoureh (Fig. 3).

We deployed Panthera® IV and V (New York, NY 10018, USA) and Cuddeback Capture Model 1125 (Non Typical, Inc., Park Falls, WI, USA), both working with white Xenon flashes with 20 to 30 seconds delay between consecutive pictures at night. They were mounted on trees or placed in rock piles, approximately 40 cm off the ground. They were inspected every five to 10 days to ensure their functionality and to save the pictures of the memory card on a portable device.

### Data preparation

**Figure 5:**
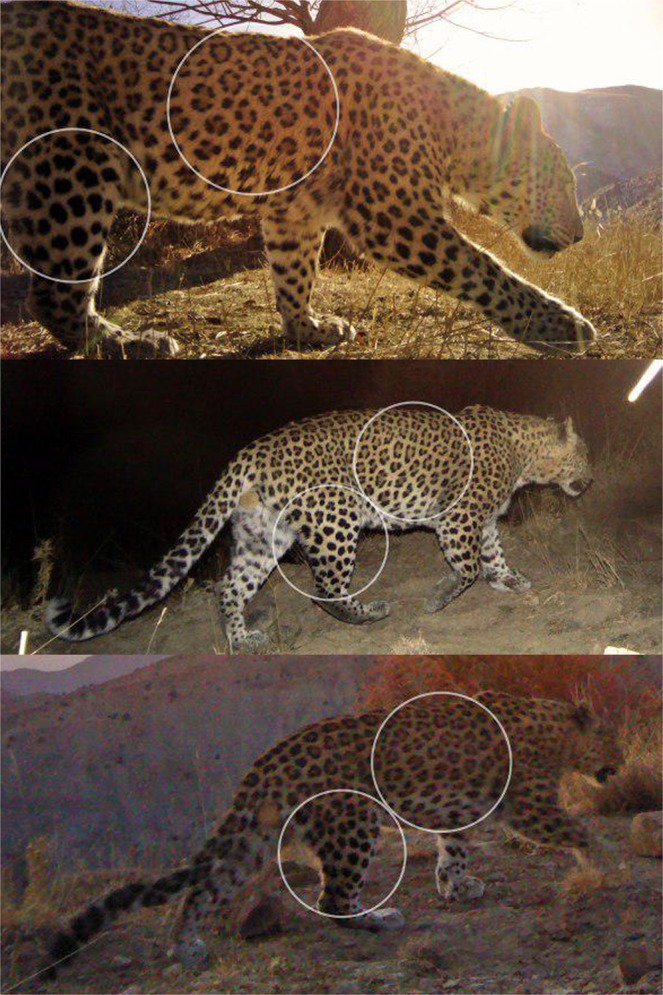
Individual identification of leopards using their unique rosette patterns. Left panel shows three adult male leopards photo-captured in Sarigol National Park, northeastern Iran. The inset circular panels show example portions with distinct rosette patterns clearly visible (© FLF/IranDoE).

The identity of leopards was determined by the unique rosette patterns on their pelage, independently by two researchers (PB and MSF, Fig. 5). Sex was distinguished where possible from sex-specific cues, such as visible genitalia or the presence of young. Because adult and sub-adult animals cannot be distinguished with certainty from pictures, we estimated the density of “independent leopard”, hence all individuals except dependent cubs.

Both left and right flanks were used to identify individual leopards and develop bilateral photographic encounter histories. When natural markings, including leopards’ rosette patterns, are bilaterally asymmetrical, matching photographs to individuals can be difficult when a single camera trap is deployed per station^28^. Therefore, we used an approach for simultaneously modeling bilateral photo-identification records in the context of capture-recapture^28–30^. Accordingly, the true encounter history of an individual is treated as a realization from a latent (unobserved) multinomial process. The observed data (right-flank, left-flank, or both-flank detections) are then dependent on the true underlying (but unobserved) encounter histories. In this manner, left-sided detection histories are allowed to be matched with right-sided detections histories from the same individual while properly accounting for uncertainty about the true number of distinct animals observed in the study^28,66^. Sometimes individuals are simultaneously photographed on both flanks by camera traps, during capture operations, or by visiting tourists; for these individuals, there is no uncertainty in their encounter history and they can thus be considered “known” with full identity. We considered 7, 4 and 21 individuals as bilaterally known from Salouk, Sarigol and Tandoureh, respectively.

For each trap location, we created an observed encounter history matrix with rows corresponding to individuals and columns corresponding to sampling occasions. Accordingly, we discarded all but one capture of the same individual taken at the same camera station within each sampling interval. As the detection data were collected from single-camera stations, we selected data type as “sometimes” because simultaneous left- and right-sided encounters for partially identified individuals were not always possible^28,29^. Hence, the entries in our observed encounter history matrix could consist of encounter type 0 (absence), 1 (left flank detection), 2 (right flank detection), 3 (non-simultaneous detection of both flanks) and 4 (simultaneous detection of both flanks).

While both left- and right-sided photographic encounters were used to estimate density and associated parameters, only the total number of unique individuals detected from right-flank encounters (due to detecting higher number of individuals comparing to left flanks) were used for calculating sex composition, number of families and variability in the number of detections between sexes, areas and sampling types.

### State-space process model

We used a hierarchical model of the temporary emigration phenomenon, composed of an explicit state-space process model and an observation model^53^. The animal population size and their respective central locations (“home-range centres”) are assumed to follow a (homogeneous) Poisson point process^27^ within the available habitat of each study area.

The state-space was described by equally spaced points in a regular grid, with a mesh size of 1 km^2^. A buffer was plotted around the trap array to incorporate individuals with activity centres outside of the trapping area, but whose movement range extends into the sampling area^53^. We applied a buffer of 40 km around a homogenous distribution of potential home-range centres, corresponding to the maximum distances between farthest locations of resident GPS collared leopards in Tandoureh^49^. We extended the buffer to 100 km in Tandoureh, to account for occasional dispersal to Turkmenistan^49^. A habitat mask was created in Quantum GIS^67^ by excluding all non-habitat areas such as villages, farmlands, and non-mountainous areas from the state space based on GPS relocation data of collared resident leopards^49^ and our field knowledge (Supplementary Fig. S1). We are confident that our the mask was large enough to avoid mask truncation bias^68^.

### Abundance and density estimation

We used the package ‘multimark’ version 2.1.0^29^ in the programme *R* version 3.3.3^69^ to fit spatially-explicit population abundance models for closed capture-mark-recapture data. The Bayesian spatial capture-recapture models in multimark accounts for the set of latent encounter histories that are feasible given the observed left- and right-sided partial identity encounter histories^28–30^ and are fitted using Markov chain Monte Carlo (MCMC). The *multimarkClosedSCR*() function in multimark implements a model that essentially combines the spatial capture-recapture model of Royle *et al*.^70^, the semi-complete data likelihood approach of King *et al*.^71^, and the multiple-mark models of Bonner & Holmberg^30^ and McClintock *et al*.^28^. Unlike the spatial partial identity model of Augustine *et al*.^72^, multimark does not rely on data augmentation for unobserved individuals and can therefore be less computationally demanding for larger populations. Model fits for Salouk and Tandoureh included 3 MCMC chains, 20,000 iterations in the adaptive phase, 440,000 iterations in the sampling phase, and 40,000 iterations for burn-in. For Sarigol, model fits included 3 chains, 120,000 adaptive iterations, 1,440,000 iterations in the sampling phase, and 240,000 iterations burin-in. Initial values for each chain were randomly drawn from the default diffuse (or “uninformative”) priors for each parameter. We assessed MCMC convergence by visually inspecting trace plots for each monitored parameter. We also calculated Gelman-Rubin-Brooks multivariate diagnostics^73^ and effective sample sizes to assess the convergence of MCMC samples and the adequacy of the MCMC chain length for all parameters using R package ‘coda’ version 0.19–2^74^. Chain lengths were selected to achieve point estimates of the multivariate potential scale reduction factor near 1 and effective sample sizes >4000^29^.

While exploring the feasible set of latent encounter histories, the parameters and latent variables to be estimated by multimark include *β*^*p*^, *N*, *D*, *σ*, *α*, *δ*, *p*, *c* and *ψ*. *β*^*p*^ is a cloglog-scale intercept terms for detection probability, *N* is population abundance, *D* is population density, *σ* represents the cloglog-scale distance term for the half-normal detection function, *α* is the conditional probability of a simultaneous type 1 and type 2 encounter (given both mark types detected), and *δ* is the conditional probability of a type 1 (left flank) or type 2 (right flank) encounter (given detection). *p* and *c* refer to the probabilities of capture and recapture, respectively^29^, and are derived using the cloglog link function^70^. *ψ* denotes the probability that a randomly selected individual from the *M* observed individuals belongs to the *n* unique individuals encountered at least once^75^. Individual activity centres and the log posterior density were also monitored.

We specified four models for detection probability (*mod*.*p*) using linear model formulas, including no effects (*mod*.*p* = *˜*1), shorthands for time variation (*mod*.*p* = *˜time*), temporal trends (*mod*.*p* = *˜Time*) and trap-specific behavioral response in detection probability to first capture (*mod*.*p* = *˜c*). We also modeled the effect of camera trap placement (water versus trail) on detection probability in Tandoureh (*mod*.*p* = *˜Placement*), including an additive model of camera trap placement and behavioral response to first capture (*mod*.*p* = *˜c* + *Placement*) and an interaction model of camera trap placement and behavioral response to first capture (*mod*.*p* = ˜c*Placement). We assumed constant *δ*_1_ = *δ*_2_ for all models because type 1 (left flank) and type 2 (right flank) encounters arise from a very similar process^29^.

After fitting the complete set of models for each study area, we performed Bayesian multimodel inference based on Barker and Link^76^ using the *multimodelClosedSCR*() function in multimark. For each study area, we fitted 3 chains each consisting of 110000 iterations with a burn-in of 10000 iterations. We reported posterior model probabilities as well as the model-averaged marginal posterior means, standard deviations and 95% credible intervals for monitored parameters.

Because sex was not determined for all encountered individuals, we were unable to include sex as a covariate in our multimark analyses. However, based on the subset of observed encounter histories for which sex was determined, we examined the effect of sex on *δ*, *p* and *c* by fitting separate multimark models for males and females including only those covariates from models which received >0% of the posterior model weight for each area as *mod*.*p* = *~c* + *Time* (Sarigol and Salouk) and *mod*.*p* = *˜c* + *Placement* (Tandoureh).

### Effects of sampling design in Tandoureh

We also used a Negative Binomial regression (implemented in the ‘MASS’ package^77^) to explore variation in detection frequency across the three areas. We also tested for goodness-of-fit for evaluating the assumption of no dispersion of residuals in the regression model with a chi-square test based on the residual deviance and degrees of freedom.

We then fitted generalized linear mixed models (GLMM) with a Poisson error distribution using the ‘lme4’ package^78^ for estimating maximum likelihood of two interactive and additive models between age/sex groups and sampling type on detection frequency. Leopard identity was included as a random effect. We checked the models for over-dispersion (i.e. the ratio of residual deviance to degrees of freedom). Also, normal distribution of residuals and homoscedasticity were checked for fitted models. The significance of terms in the final model was assessed using log-likelihood ratio tests for comparing the goodness of fit between models. We then used least-squares means to predict the effects of sampling type on each different age/sex group from the final GLMM model using ‘lsmeans’ package^79^. Finally, we used Fisher’s exact test to examine the null hypothesis that the proportion of different age and sex classes detected are independent of whether a camera is placed on water or trail in Tandoureh.

## Supplementary information

Supplementary Information

## Data Availability

R scripts and datasets analysed during the current study are available on Figshare with the generated link as (https://figshare.com/s/e2f2cb7632671c440a3a).
