# Supplementary material for: A paradox of local abundance amidst regional rarity: the value of montane refugia for Persian leopard conservation
Source: Sci Rep. 2019 Oct 11;9:14622. doi: 10.1038/s41598-019-50605-2 (PMC6788991; doi:10.1038/s41598-019-50605-2)
Supplement: Supplementary file 1 — Supplementary Information [file 41598_2019_50605_MOESM1_ESM.docx]

**Supplementary Information**

**A paradox of local abundance amidst regional rarity: the value of montane refugia for Persian leopard conservation**

Mohammad S. Farhadinia^1 2^ *, Brett McClintock^3^, Paul J. Johnson^1^, Pouyan Behnoud^2^, Kaveh Hobeali^2^, Peyman Moghadas^2^, Luke T.B. Hunter^3 4^ and David W. Macdonald^1^

^1^ Wildlife Conservation Research Unit, University of Oxford, Tubney House, Oxfordshire, OX13 5QL, Oxford, UK

^2^ Future4Leopards Foundation, Tehran, Iran

^3^ National Marine Mammal Laboratory, Alaska Fisheries Science Center, NOAA‐NMFS, 7600 Sand Point Way NE, Seattle, Washington, 98115, USA

^4^ School of Life Sciences, Westville Campus, University of KwaZulu-Natal, Durban, South Africa

* Corresponding author email: [mohammad.farhadinia@zoo.ox.ac.uk](mailto:mohammad.farhadinia@zoo.ox.ac.uk) (ORCID ID: 0000-0002-5385-6254/ T +44 (0)1865 611 116)

Supplementary Figure S1. Posterior density of activity centers within the available habitat in A) Sarigol, B) Salouk and C) Tandoureh. Each black dot represents a camera trap station.

1. *Sarigol*


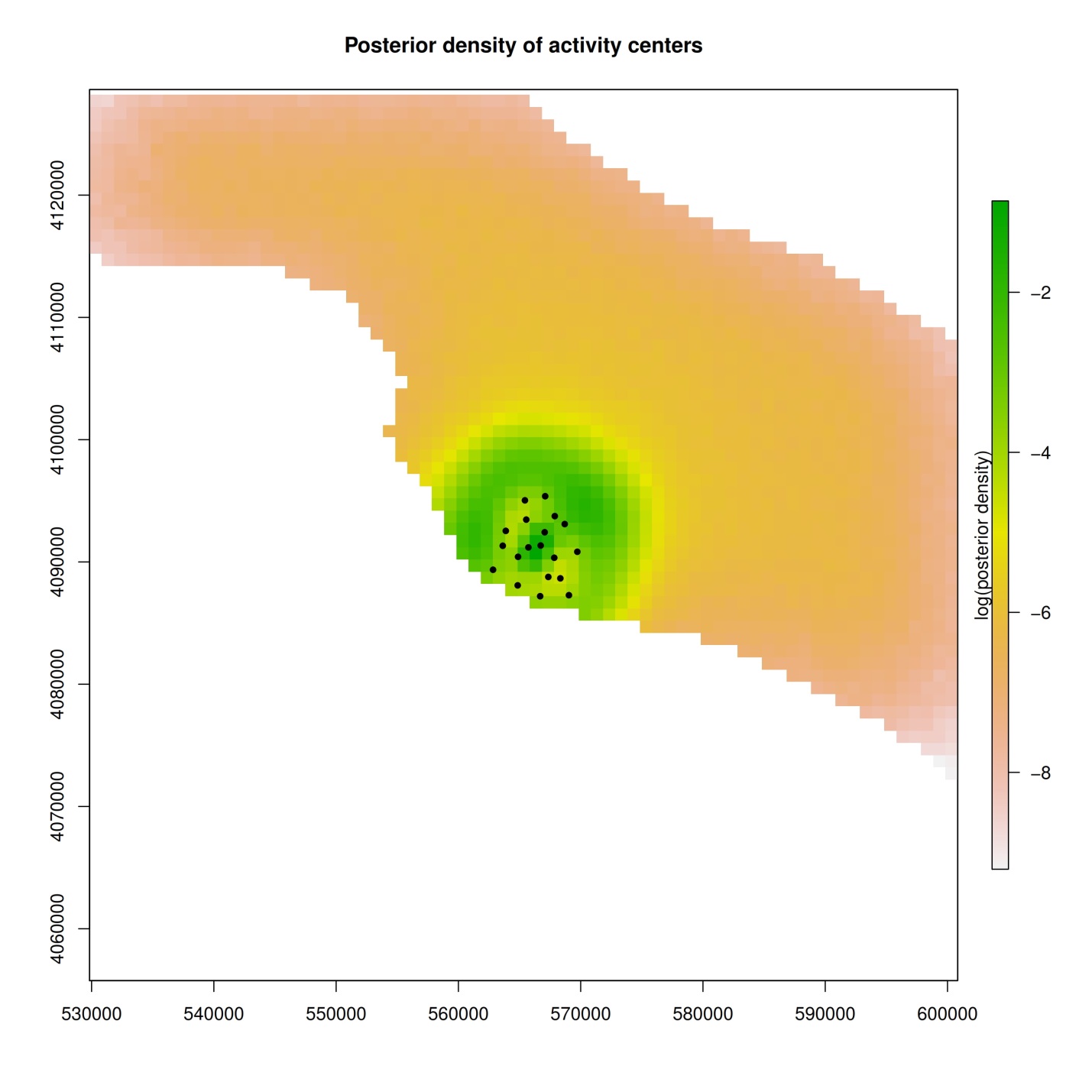


1. *Salouk*


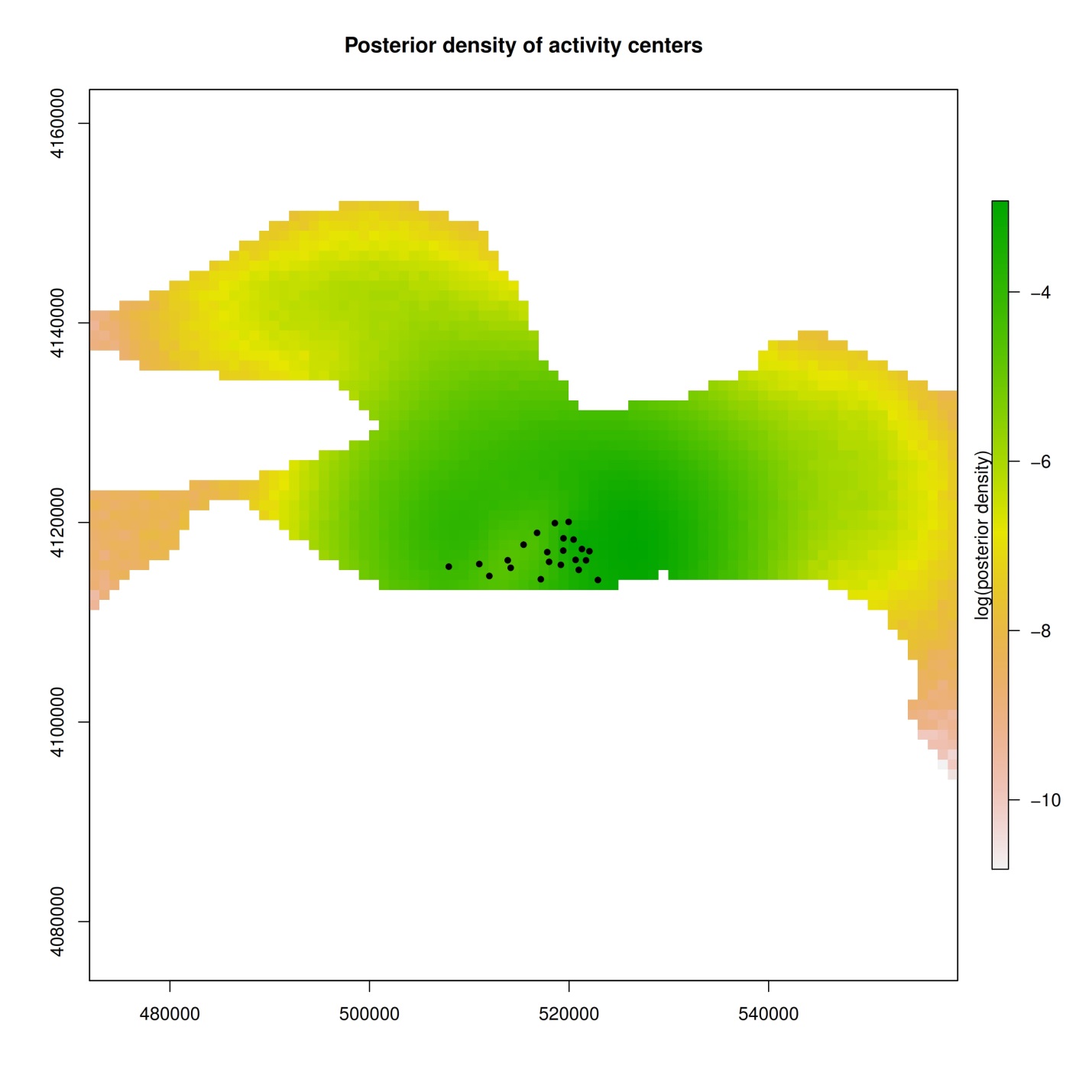


1. *Tandoureh*


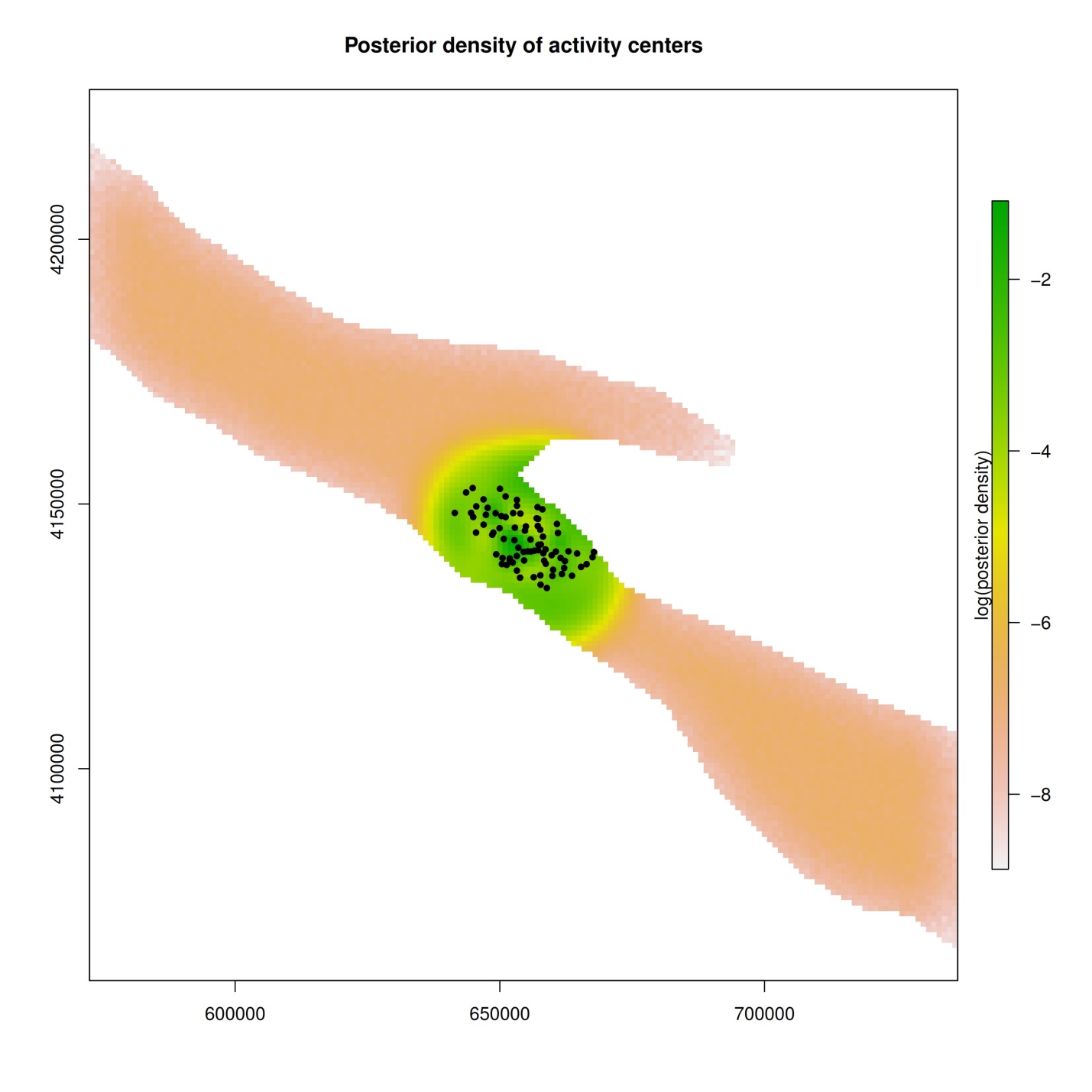


Supplementary Table S1. A review of density estimates of leopards across their global range, using photographic capture-recapture methodology, published as of 1 January 2018.

| **Number** | **Country** | **Continent** | **Subspecies** | **Study area** | **Density (individual/100 km^2^)** | **Cub/Total population ratio** | **Study** |
| --- | --- | --- | --- | --- | --- | --- | --- |
| 1 | Cambodia | Asia | *delacouri* | Mondulkiri Protected Forest | 3.6 | 0 | 1 |
| 2 | Malaysia | Asia | *delacouri* | Taman Negara | 3 | NA | 2 |
| 3 | India | Asia | *fusca* | Cilla forest | 14.9 | NA | 3 |
| 4 | Bhutan | Asia | *fusca* | Jigme Singye Wangchuck National Park | 1.04 | NA | 4 |
| 5 | India | Asia | *fusca* | Pakke Tiger Reserve, Arunachal Pradesh | 2.82 | NA | 5 |
| 6 | India | Asia | *fusca* | Akole | 4.8 | NA | 6 |
| 7 | India | Asia | *fusca* | Sariska (2007) | 8.4 | NA | 7 |
| 8 | India | Asia | *fusca* | Sariska (2008) | 7.4 | NA | 7 |
| 9 | India | Asia | *fusca* | Sariska (2009) | 5.2 | NA | 7 |
| 10 | India | Asia | *fusca* | Sariska (2010) | 2.3 | NA | 7 |
| 11 | India | Asia | *fusca* | Sariska (2011) | 5.8 | NA | 7 |
| 12 | Nepal | Asia | *fusca* | Bhabhar of Terai Arc | 3.7 | 0 | 8 |
| 13 | India | Asia | *fusca* | Mudumalai Tiger Reserve | 13.1 | NA | 9 |
| 14 | Bhutan | Asia | *fusca* | Royal Manas National Park | 10 | NA | 10 |
| 15 | India | Asia | *fusca* | Manas | 3.4 | NA | 11 |
| 16 | India | Asia | *fusca* | Churna (1) | 8 | 0.17 | 12 |
| 17 | India | Asia | *fusca* | Churna (2) | 9.3 | 0.15 | 12 |
| 18 | India | Asia | *fusca* | Kamti | 7.5 | 0 | 12 |
| 19 | India | Asia | *fusca* | Lagda | 7.3 | 0 | 12 |
| 20 | Sri Lanka | Asia | *kotiya* | Horton Plains National Park | 11.7 | 0.11 | 13 |
| 21 | Sri Lanka | Asia | *kotiya* | Ruhuna (Yala) | 12.3 | NA | 13 |
| 22 | Sri Lanka | Asia | *kotiya* | Ruhuna (Yala) | 14.7 | NA | 13 |
| 23 | Sri Lanka | Asia | *kotiya* | Ruhuna (Yala) | 10 | NA | 13 |
| 24 | Sri Lanka | Asia | *kotiya* | Ruhuna (Yala) | 11.4 | NA | 13 |
| 25 | Russia | Asia | *orientalis* | Primorski Krai (N unit) | 1.1 | 0 | 14 |
| 26 | Russia | Asia | *orientalis* | Primorski Krai (S unit) | 1.03 | 0 | 14 |
| 27 | China | Asia | *orientalis* | Northeastern China | 0.05 | 0.09 | 15 |
| 28 | South Africa | Africa | *pardus* | Mkhuze (2005) | 11.11 | NA | 16 |
| 29 | South Africa | Africa | *pardus* | Mkhuze (2008) | 10.76 | NA | 16 |
| 30 | South Africa | Africa | *pardus* | Non-PAs (2005) | 2.49 | NA | 16 |
| 31 | South Africa | Africa | *pardus* | Phinda (2005) | 7.17 | NA | 16 |
| 32 | South Africa | Africa | *pardus* | Phinda | 6.97 | 0.07 | 17 |
| 33 | South Africa | Africa | *pardus* | Phinda (2005) | 7.17 | 0.07 | 18 |
| 34 | South Africa | Africa | *pardus* | Phinda (2007) | 9.42 | 0.07 | 18 |
| 35 | South Africa | Africa | *pardus* | Phinda (2009) | 11.21 | 0.07 | 18 |
| 36 | Namibia | Africa | *pardus* | Waterberg Plateau Park | 1 | NA | 19 |
| 37 | Namibia | Africa | *pardus* | Waterberg Plateau Farm | 3.6 | NA | 19 |
| 38 | Gabon | Africa | *pardus* | Central Gabon (2) | 2.69 | NA | 20 |
| 39 | Gabon | Africa | *pardus* | Central Gabon (3) | 4.58 | NA | 20 |
| 40 | Gabon | Africa | *pardus* | Central Gabon (4) | 12.08 | NA | 20 |
| 41 | South Africa | Africa | *pardus* | Lapalala, Waterberg Biosphere | 5.35 | NA | 21 |
| 42 | South Africa | Africa | *pardus* | Farming matrix, Waterberg Biosphere | 6.59 | NA | 21 |
| 43 | South Africa | Africa | *pardus* | Welgevonden, Waterberg Biosphere | 4.56 | NA | 21 |
| 44 | South Africa | Africa | *pardus* | Zululand Rhino Reserve | 7 | 0.25 | 22 |
| 45 | South Africa | Africa | *pardus* | N’wanetsi concession, Kruger | 12.7 | NA | 23 |
| 46 | South Africa | Africa | *pardus* | Western Shore | 8.42 | 0 | 24 |
| 47 | South Africa | Africa | *pardus* | Eastern Shore | 7.4 | 0 | 24 |
| 48 | South Africa | Africa | *pardus* | Tembe | 4.8 | 0 | 24 |
| 49 | South Africa | Africa | *pardus* | Ndumo | 1.6 | 0.4 | 24 |
| 50 | South Africa | Africa | *pardus* | Phinda | 3.4 | 0.12 | 25 |
| 51 | South Africa | Africa | *pardus* | Phinda | 3.47 | 0.13 | 25 |
| 52 | Zimbabwe | Africa | *pardus* | Kwalusi (baited) | 6.1 | 0.12 | 26 |
| 53 | Zimbabwe | Africa | *pardus* | Mazunga (baited) | 4.6 | 0.12 | 26 |
| 54 | Zimbabwe | Africa | *pardus* | Kwalusi (unbaited) | 5.5 | 0 | 26 |
| 55 | Zimbabwe | Africa | *pardus* | Mazunga (unbaited) | 2.8 | 0 | 26 |
| 56 | Kenya | Africa | *pardus* | Mpala Ranch, Laikipia | 12.03 | 0 | 27 |
| 57 | Zambia | Africa | *pardus* | CD 2013, ESA | 6.07 | NA | 28 |
| 58 | Zambia | Africa | *pardus* | HD 2013, ESA | 4.2 | NA | 28 |
| 59 | Zambia | Africa | *pardus* | CD 2014, ESA | 4.2 | NA | 28 |
| 60 | Zambia | Africa | *pardus* | HD 2014, ESA | 5.87 | NA | 28 |
| 61 | Zambia | Africa | *pardus* | CD 2012, WSA | 10.15 | NA | 28 |
| 62 | Zambia | Africa | *pardus* | CD 2013, WSA | 8.3 | NA | 28 |
| 63 | Zambia | Africa | *pardus* | HD 2013, WSA | 7.16 | NA | 28 |
| 64 | Zambia | Africa | *pardus* | CD 2014, WSA | 7.16 | NA | 28 |
| 65 | Zambia | Africa | *pardus* | HD 2014, WSA | 9.72 | NA | 28 |
| 66 | South Africa | Africa | *pardus* | Soutpansberg Mountains | 10.41 | 0.29 | 29 |
| 67 | Iran | Asia | *saxicolor* | Golestan | 2.63 | 0.05 | 30 |
| 68 | Iran | Asia | *saxicolor* | Bamou | 1.87 | 0.14 | 31 |
| 69 | Iran | Asia | *saxicolor* | Tandoureh | 5.57 | 0.19 | This study |
| 70 | Iran | Asia | *saxicolor* | Sarigol | 8.86 | 0.09 | This study |
| 71 | Iran | Asia | *saxicolor* | Salouk | 3.10 | 0.19 | This study |
| 72 | Armenia | Asia | *saxicolor* | Nuvadi | 0.34 | 0 | 32 |

Supplementary Table S2. Model-averaged posterior mean, standard deviations and 95% credible intervals (CI) for models including only those covariates which received >0% of the posterior model weight for each area in northeastern Iran. *p* and *c* = probabilities of capture and recapture respectively. To investigate the effect of sex, the best performing model for each area as mod.p=~c+Time (Sarigol and Salouk) and mod.p=˜c+Placement (Tandoureh) were run for each sex separately.

| **Parameter** | **Posterior mean** | **SD** | **CI** |
| --- | --- | --- | --- |
| **Sarigol NP** |  |  |  |
| *p* _first occasion Male_ | 0.40 | 0.21 | 0.13-0.98 |
| *p* _first occasion Female_ | 0.27 | 0.14 | 0.07-0.60 |
| *p* _last occasion Male_ | 0.08 | 0.10 | 0.01-0.33 |
| *p* _last occasion Female_ | 0.17 | 0.15 | 0.02-0.58 |
| *c* _second occasion Male_ | 0.36 | 0.24 | 0.08-0.99 |
| *c* _second occasion Female_ | 0.37 | 0.16 | 0.123-0.73 |
| *c* _last occasion Male_ | 0.08 | 0.10 | 0.01-0.39 |
| *c* _last occasion Female_ | 0.24 | 0.14 | 0.06-0.58 |
| **Salouk NP & PA** |  |  |  |
| *p* _first occasion Male_ | 0.55 | 0.28 | 0.13-1.00 |
| *p* _first occasion Female_ | 0.12 | 0.10 | 0.04-0.39 |
| *p* _last occasion Male_ | 0.13 | 0.16 | 0.01-0.63 |
| *p* _last occasion Female_ | 0.02 | 0.03 | 0.003-0.07 |
| *c* _second occasion Male_ | 0.51 | 0.28 | 0.10-0.99 |
| *c* _second occasion Female_ | 0.21 | 0.15 | 0.05-0.64 |
| *c* _last occasion Male_ | 0.13 | 0.16 | 0.01-0.64 |
| *c* _last occasion Female_ | 0.04 | 0.06 | 0.01-0.16 |
| **Tandoureh NP** |  |  |  |
| *p* _Water Male_ | 0.05 | 0.01 | 0.03-0.07 |
| *p* _Water Female_ | 0.08 | 0.02 | 0.04-0.12 |
| *p* _Trail Male_ | 0.03 | 0.06 | 0.02-0.04 |
| *p* _Trail Female_ | 0.03 | 0.007 | 0.01-0.04 |
| *c* _Water Male_ | 0.34 | 0.05 | 0.25-0.43 |
| *c* _Water Female_ | 0.47 | 0.07 | 0.35-0.61 |
| *c* _Trail Male_ | 0.21 | 0.04 | 0.15-0.29 |
| *c* _Trail Female_ | 0.19 | 0.04 | 0.12-0.29 |

### References

1. Gray, T. N. E. & Prum, S. Leopard Density in Post-Conflict Landscape, Cambodia: Evidence From Spatially Explicit Capture-Recapture. *J. Wildl. Manage.* **76**, 163–169 (2012).

2. Hedges, L. *et al.* Melanistic leopards reveal their spots: infrared camera traps provide a population density estimate of leopards in Malaysia. *J. Wildl. Manage.* **79**, 846–853 (2015).

3. Harihar, A., Pandav, B. & Goyal, S. P. Density of leopards (Panthera pardus) in the Chilla Range of Rajaji National Park, Uttarakhand, India. *Mammalia* **73**, 68–71 (2009).

4. Wang, S. W. & MacDonald, D. W. The use of camera traps for estimating tiger and leopard populations in the high altitude mountains of Bhutan. *Biol. Conserv.* **142**, 606–613 (2009).

5. Selvan, K. M., Lyngdoh, S., Habib, B. & Gopi, G. V. Population density and abundance of sympatric large carnivores in the lowland tropical evergreen forest of Indian Eastern Himalayas. *Mamm. Biol.* **79**, 254–258 (2014).

6. Athreya, V., Odden, M., Linnell, J. D. C., Krishnaswamy, J. & Karanth, U. Big cats in our backyards: persistence of large carnivores in a human dominated landscape in India. *PLoS One* **8**, 1–8 (2013).

7. Mondal, K., Sankar, K., Qureshi, Q., Gupta, S. & Chourasia, P. Estimation of population and survivorship of leopard Panthera pardus (Carnivora: Felidae) through photographic capture–recapture sampling in western India. *World J. Zool.* **7**, 30–39 (2012).

8. Thapa, K. *et al.* Leopard Panthera pardus fusca density in the seasonally dry, subtropical forest in the Bhabhar of Terai Arc, Nepal. *Adv. Ecol.* **2014**, (2014).

9. Kalle, R., Ramesh, T., Qureshi, Q. & Sankar, K. Density of tiger and leopard in a tropical deciduous of Mudumalai Tiger Reserve, southern India, as estimated using photographic capture-recapture sampling. *Acta Theriol. (Warsz).* **56**, 335–342 (2011).

10. Goldberg, J. F. *et al.* Examining temporal sample scale and model choice with spatial capture-recapture models in the common leopard Panthera pardus. *PLoS One* **10**, e0140757 (2015).

11. Borah, J. *et al.* Abundance and density estimates for common leopard Panthera pardus and clouded leopard Neofelis nebulosa in Manas National Park, Assam, India. *Oryx* **48**, 149–155 (2014).

12. Edgaonkar, A. Ecology of the Leopard (Panthera pardus) in Bori wildlife sanctuary and Satpura national park, India. (2008).

13. Kittle, A. M., Watson, A. C. & Fernando, T. S. P. The ecology and behavior of a protected area Sri Lankan leopard (Panthera pardus kotiya) population. *Trop Ecol* **57**, 71–86 (2017).

14. Kostyria, A. V, Skorodelov, A. S., Miquelle, D. G., Aramilev, V. V & McCullough, D. *Results of camera trap survey of Far Eastern Leopard population in southwest Primorski Krai, winter 2002-2003*. (Wildlife Conservation Society & ISUNR, 2003).

15. Jiang, G. *et al.* New hope for the survival of the Amur leopard in China. *Sci. Rep.* **5**, 15475 (2015).

16. Balme, G. A., Slotow, R. & Hunter, L. T. B. Edge effects and the impact of non-protected areas in carnivore conservation: leopards in the Phinda? Mkhuze Complex, South Africa. *Anim. Conserv.* **13**, 315–323 (2010).

17. Balme, G. A., Hunter, L. T. B. & Slotow, R. Evaluating methods for counting cryptic carnivores. *J.Wildl.Manage.* **73**, 433–441 (2009).

18. Balme, G. A., Slotow, R. & Hunter, L. T. B. Impact of conservation interventions on the dynamics and persistence of a persecuted leopard (Panthera pardus) population. *Biol. Conserv.* **142**, 2681–2690 (2009).

19. Stein, A. B., Fuller, T. K., DeStefano, S. & Marker, L. Leopard population and home range estimates in north-central Namibia. *Afr. J. Ecol.* **49**, 383–387 (2011).

20. Henschel, P. The conservation biology of the leopard Panthera pardus in Gabon: Status, threats and strategies for conservation. *MathematischNaturwissenschaftlichen Fakultäten* **PhD thesis**, (2008).

21. Swanepoel, L. H., Somers, M. J. & Dalerum, F. Density of leopards Panthera pardus on protected and non-protected land in the Waterberg Biosphere, South Africa. *Wildlife Biol.* **21**, 263–268 (2015).

22. Chapman, S. & Balme, G. A. An estimate of leopard population density in a private reserve in KwaZulu-Natal, South Africa, using camera-traps and capture-recapture models. *South African J. Wildl. Res.* **40**, 114–120 (2010).

23. Maputla, N. W., Chimimba, C. T. & Ferreira, S. M. Calibrating a camera trap–based biased mark–recapture sampling design to survey the leopard population in the N’wanetsi concession, Kruger National Park, South Africa. *Afr. J. Ecol.* **51**, 422–430 (2013).

24. Ramesh, T., Kalle, R., Rosenlund, H. & Downs, C. T. Low leopard populations in protected areas of Maputaland: a consequence of poaching, habitat condition, abundance of prey, and a top predator. *Ecol. Evol.* **7**, 1964–1973 (2017).

25. Braczkowski, A. R. *et al.* Scent lure effect on camera-trap based leopard density estimates. *PLoS One* **11**, e0151033 (2016).

26. du Preez, B., Loveridge, A. J. & Macdonald, D. W. To bait or not to bait: A comparison of camera-trapping methods for estimating leopard (Panthera pardus) density. *Biol. Conserv.* **176**, 153–161 (2014).

27. O’Brien, T. G. & Kinnaird, M. F. Density estimation of sympatric carnivores using spatially explicit capture-recapture methods and standard trapping grid. *Ecol. Appl.* **21**, 2908–2916 (2011).

28. Rosenblatt, E. *et al.* Effects of a protection gradient on carnivore density and survival: an example with leopards in the Luangwa valley, Zambia. *Ecol. Evol.* **6**, 3772–3785 (2016).

29. Grey, J. N. C., Kent, V. T. & Hill, R. A. Evidence of a high density population of harvested leopards in a montane environment. *PLoS One* **8**, e82832 (2013).

30. Hamidi, A. K. *et al.* Camera trap study of Persian leopard in Golestan National Park, Iran. *Cat News* **60**, 12–14 (2014).

31. Ghoddousi, A., Hamidi, A. K., Ghadirian, T., Ashayeri, D. & Khorozyan, I. The status of the endangered Persian leopard Panthera pardus saxicolor in Bamu National Park, Iran. *Oryx* **44**, 551–557 (2010).

32. Khorozyan, I., Malkhasyan, A. G. & Abramov, A. V. Presence-absence survey of prey and their use in predicting leopard (Panthera pardus) densities: a case study from Armenia. *Integr. Zool.* **3**, 322–332 (2008).
